# Genetic Diversity of Rotaviruses Circulating in Pediatric Patients and Domestic Animals in Thailand

**DOI:** 10.3390/tropicalmed8070347

**Published:** 2023-06-29

**Authors:** Nutthawadee Jampanil, Kattareeya Kumthip, Niwat Maneekarn, Pattara Khamrin

**Affiliations:** 1 Department of Microbiology, Faculty of Medicine, Chiang Mai University, Chiang Mai 50200, Thailand; 2 Emerging and Re-Emerging Diarrheal Viruses Cluster, Chiang Mai University, Chiang Mai 50200, Thailand

**Keywords:** rotavirus, diarrhea, children, Thailand

## Abstract

Rotavirus A is a highly contagious virus that causes acute gastroenteritis in humans and a wide variety of animals. In this review, we summarized the information on rotavirus described in the studies in the last decade (2008 to 2021) in Thailand, including the prevalence, seasonality, genetic diversity, and interspecies transmission. The overall prevalence of rotavirus infection in humans ranged from 15–33%. Rotavirus infection was detected throughout the year and most frequently in the dry and cold months, typically in March. The diversity of rotavirus genotypes varied year to year and from region to region. From 2008 to 2016, rotavirus G1P[8] was detected as the most predominant genotype in Thailand. After 2016, G1P[8] decreased significantly and other genotypes including G3P[8], G8P[8], and G9P[8] were increasingly detected from 2016 to 2020. Several uncommon rotavirus strains such as G1P[6], G4P[6], and G3P[10] have also been occasionally detected. In addition, most studies on rotavirus A infection in animals in Thailand from 2011 to 2021 reported the detection of rotavirus A in piglets and canine species. It was reported that rotavirus could cross the host species barrier between humans and animals through interspecies transmission and genetic reassortment mechanisms. The surveillance of rotavirus infection is crucial to identify the trend of rotavirus infection and the emergence of novel rotavirus genotypes in this country. The data provide information on rotavirus infection and the diversity of rotavirus genotypes circulating in the pre-vaccination period, and the data will be useful for the evaluation of the effectiveness of rotavirus vaccine implementation in Thailand.

## 1. Introduction

Rotavirus (RV) is the main causative agent of acute viral gastroenteritis in infants and young children worldwide and in a wide variety of animal species [1]. Globally, rotavirus infection accounts for an estimated 30–50% of childhood diarrheal hospitalizations and is associated with >200,000 deaths annually in children under five years of age, with the mortality rate being greatest in South Asia and sub-Saharan Africa [2,3,4]. Rotavirus is transmitted via the fecal-oral route. Certainly, contaminated food or water, unsanitary surfaces, and poor hygiene are the risk factors for rotavirus infection [5]. The symptoms of rotavirus infection typically include diarrhea, vomiting, fever, and abdominal pain. Currently, there is no specific treatment for rotavirus infection, and in most cases, it is a self-limited disease with spontaneous recovery within three to eight days [6]. The best way to prevent rotavirus infection is vaccination. Rotavirus live-attenuated vaccines that have been prequalified by WHO and have been licensed globally/nationally are Rotarix (RV1), RotaTeq (RV5), ROTAVAC, and ROTASIL [7]. Although rotavirus vaccines for infants have been introduced in over 100 countries, rotavirus-associated mortality remains high in low-income countries [7,8]. In addition, both Rotarix and RotaTeq vaccines have been incorporated into Thailand’s national childhood immunization program since 2020 [9]. 

Rotavirus is classified into nine species (A, B, C, D, F, G, H, I, and J) [10]. Among these, rotavirus A (RVA) is the most widespread and medically significant species worldwide [6,11]. The genetic diversity of rotavirus is driven by interspecies transmission and genetic reassortment events, which are the important mechanisms of rotavirus evolution [12]. Humans can be infected by rotavirus of animal origins through direct transmission or by the exchange of one or more genome segments among human and animal rotaviruses [13]. Several studies have demonstrated that human rotavirus strains have originated from animal strains of independent ancestors [12]. In addition, some of the uncommon rotavirus strains that have been detected in humans are derived from viruses transmitted between humans and animals [14].

In Thailand, several studies of rotavirus epidemiology have been conducted over the past four decades. The epidemiology of rotavirus infection in Thailand from 1977 to 1996 and 2000 to 2011 has been reviewed previously by Maneekarn et al. [15,16]. The present review article summarized the information on rotavirus prevalence and their genotypes, the temporal distribution patterns, and described the rotavirus interspecies transmissions among humans and animals, which were detected in Thailand from 2008 to 2020 and have not been included in the previous reviews.

## 2. Virus Biology and Classification

### 2.1. Rotavirus Biology

Rotaviruses were first discovered in rectal swabs of monkeys and intestinal epithelium tissue biopsy of mice by electron microscopy in the 1950s and 1960s, respectively [17]. In 1973, Ruth Bishop and coworkers first described rotavirus as a human pathogen in tissue biopsy of duodenum epithelial cells and stools from nine children with acute gastroenteritis [18]. Later, rotavirus was detected in large quantities in stool samples from children hospitalized with acute non-bacterial gastroenteritis by negative staining electron microscopy [19]. Under the electron microscope, the viral particle with a wheel-like appearance of approximately 70 nm in diameter was observed and designated subsequently as rotavirus (the term rotavirus is derived from the Latin word *rota*, which means wheel) [20]. Rotavirus is classified into the genus *Rotavirus* in the family *Sedoreoviridae* in the order *Reovirales* [10]. Rotavirus is a large non-enveloped virus with a size of approximately 65 to 75 nm in diameter, which consists of three concentric icosahedral capsid structures. The outer layer is composed of two capsid proteins, VP4 and VP7. The intermediate layer is comprised of a single type of VP6 protein. The internal core consists of VP2 associated with VP1 (RNA-dependent RNA polymerase) and VP3 (viral capping enzyme). The viral genome, comprised of 11 segments of double-stranded RNA (dsRNA), is packaged entirely within the internal core layer [1,21]. Each rotavirus genome segment contains one to two open reading frames with 5′- and 3′- terminal noncoding regions. The rotavirus genome encodes for six viral structural proteins (VP1-VP4, VP6, and VP7) and six nonstructural proteins (NSP1-NSP6) [1,22]. The structural proteins integrated into the virion determines host specificity and cell entry. The nonstructural proteins, constructed during infection, are involved in viral replication and pathogenesis and inhibit the innate immune response to infection [3,7]. According to the antigenicity of the intermediate layer VP6, at least nine different species of rotaviruses have been classified [10]. Among these, species A to C and H infect human and mammalian species. However, rotavirus A is the most widespread species and predominantly infects humans, especially young children [3,14].

### 2.2. Dual Classification System

The rotavirus strains have been classified by a dual classification system based on antigenic and sequence differences of the two outer capsid proteins, VP7 and VP4 proteins, into G (glycosylated) and P (protease-sensitive) genotypes, respectively [21]. Although 42 G genotypes and 58 P genotypes have been described in humans and animals worldwide [23], only a few combinations of G and P genotypes are predominantly detected in humans. The most frequently detected human rotavirus genotypes are G1P[8], G2P[4], G3P[8], G4P[8], G8P[8], G9P[8], and G12P[8] [7,24].

### 2.3. Full-Length Genome Classification System 

Due to the increasing of rotavirus sequence data, the Rotavirus Classification Working Group (RCWG) established a new rotavirus classification system based on the nucleotide sequence identities of each genome segment in the order of Gx-P[x]-Ix-Rx-Cx-Mx-Ax-Nx-Tx-Ex-Hx to represented the genotypes of VP7-VP4-VP6-VP1-VP2-VP3-NSP1-NSP2-NSP3-NSP4-NSP5/6 [25]. Within rotavirus A, at least 42 G genotypes, 58 P genotypes, 32 I genotypes, 28 R genotypes, 24 C genotypes, 24 M genotypes, 39 A genotypes, 28 N genotypes, 28 T genotypes, 32 E genotypes, and 28 H genotypes of rotaviruses have been identified from humans and several animal species [23].

### 2.4. Genotype Constellations

Two genotype constellations of rotavirus A, I1-R1-C1-M1-A1-N1-T1-E1-H1 (Wa-like) and I2-R2-C2-M2-A2-N2-T2-E2-H2 (DS-1-like), have been shown to circulate worldwide in humans [21,26]. The Wa-like strains have been demonstrated to share a common ancestor with the porcine rotavirus, whereas DS-1-like strains have been shown to have several gene segments that have a common origin with the bovine rotavirus [27]. In addition to Wa-like and DS-1-like rotaviruses, another genotype (AU-1-like), G3-P[9]-I3-R3-C3-M3-A3-N3-T3-E3-H3 is rarely detected in humans and considered to share a common ancestor with feline and canine rotavirus strains [28]. 

## 3. Molecular Epidemiology and Genetic Diversity

Rotavirus A is the main causative agent of acute gastroenteritis in infants and young children under five years of age and is the most significant pathogen associated with the mortality of children in several countries worldwide, with infection rates ranging from 30–50% [3,29]. Moreover, rotavirus infection was estimated to cause 151,714 deaths among children under five years of age in 2019, and 90% of cases were in developing or low-income countries, probably due to limited access to health care services, lack of hydration therapy, and malnutrition [3,30]. Generally, G1P[8], G2P[4], G3P[8], G4P[8], G8P[8], G9P[8], and G12P[8] are the most prevalent strains and account for over 70% of all strains circulated worldwide [13].

### 3.1. Rotavirus Prevalence and Age Distribution

**Figure 1:**
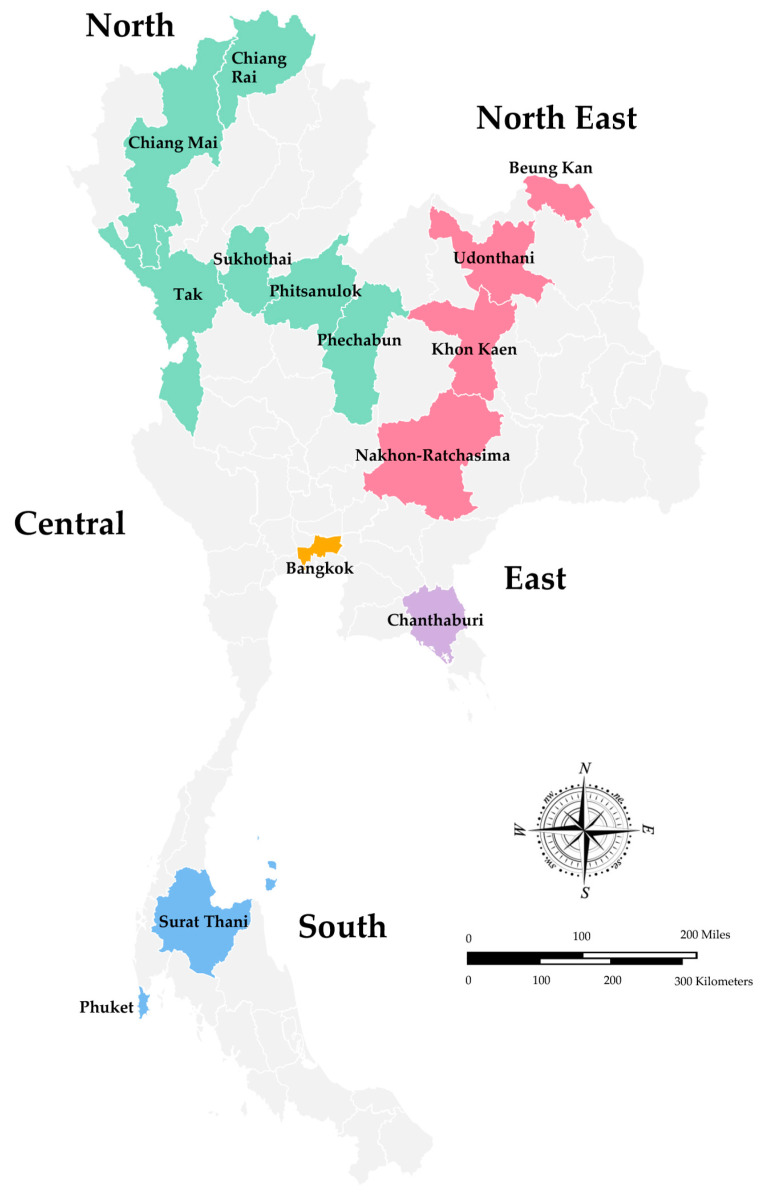
A Map of Thailand Indicates the Regions and Provinces where the Stool Samples were Collected.

In Thailand, the surveillance of rotavirus infection has been conducted in Bangkok since 1977, and subsequently, several studies have been reported from different geographical regions of Thailand, including the North, Northeast, Eastern, Central, and South regions [15,31]. The overall infection rate of rotavirus A in children hospitalized with diarrhea in Thailand, summarized from two reviews from 1977 to 2011, ranged from 22.8% to 44.5% [15,16]. However, the studies using the specimens collected from 2008 to 2010 that were published recently and have not been included in the previous reviews are also described in this review. A map of Thailand indicates the regions and provinces where the stool samples were collected is shown in Figure 1. As shown in Table 1, the study conducted in Chiang Rai, Nakhon-Ratchasima, Surat Thani, and Phitsanulok Provinces reported the prevalence of rotavirus A infection in children with acute gastroenteritis from 2008 to 2010 at 26.8% and more than 70% of the patients were younger than two years old [32]. Likewise, children hospitalized with acute gastroenteritis in Chiang Mai Province from 2010 to 2013 were infected with rotavirus 17.8%, and a high infection rate was also observed in children younger than 24 months old [33]. The study conducted in Bangkok and Khon Kaen Provinces from 2011 to 2014 revealed the prevalence of rotavirus infection at 30% [34], whereas the prevalence in Phechabun and Sukhothai provinces in 2013 and 2014 was 24% [35]. The rotavirus surveillance in Bangkok, Udonthani, Beung Kan, Phuket, Tak, and Chanthaburi Provinces from 2014 to 2016 revealed the prevalence of rotavirus infection at 27.5% in children between the age of five to ten years old [36]. The prevalence of rotavirus infection in children with acute gastroenteritis in Chiang Rai Province (Northern Thailand) was 33.7% from 2015 to 2016 [37] and dropped to 11.5% from 2018 to 2020 [38]. In addition, the prevalence of rotavirus infection in children hospitalized with diarrhea from 2018 to 2019 in Chiang Mai (Northern Thailand) was 17.9% [39], and in Bangkok (Central Thailand) from 2016 to 2019 dropped down to 15.7% [40]. Altogether, it should be noted that the prevalence of rotavirus infection in children with acute gastroenteritis in Thailand from 2011 to 2016 ranged from 24.2 to 33.7% and dropped down to the range of 11.5–15.0% from 2016 to 2020. Furthermore, it is interesting to point out that the prevalence of rotavirus infection in children hospitalized with acute gastroenteritis in Chiang Mai Province from 2010 to 2013 and 2018 to 2019 remained the same at 17.8% and 17.9%.

The global trend in the prevalence of rotavirus infection declined substantially many years ago, particularly in countries where rotavirus vaccines have been implemented [41,42,43,44]. In Thailand, RotaTeq and Rotarix vaccines were initially introduced as voluntary vaccines in 2005 and 2008, respectively [9,45]. Furthermore, in 2020, the rotavirus vaccines were incorporated into Thailand’s national childhood immunization program. Moreover, during the COVID-19 pandemic, intensive control measures on the COVID-19 pandemic have been implemented, including physical distancing, surface disinfection, and improved hand hygiene, which can potentially impact the overall occurrence of infectious diseases, including rotavirus infection [41,46]. Consequently, these factors may have influenced the prevalence of rotavirus infection among Thai children recently.

### 3.2. Seasonal Patterns of Human Rotavirus Infection 

In Thailand, several studies revealed that rotavirus in children hospitalized with acute gastroenteritis could be detected year-round with peaks in the cool and dry months (November to May) [44], as summarized in Table 1. The surveillance of rotavirus in Chiang Rai, Nakhon-Ratchasima, Surat-Thani, and Phitsanulok Provinces from 2008 to 2010 revealed that rotavirus infection reached a peak from November to April [32]. Another study in Chiang Rai Province from 2015 to 2016 reported the highest rate of rotavirus detection in March [37]. Later, the study conducted in Chiang Mai Province from 2018 to 2019 revealed the high detection rate of rotavirus from January to March [39]. Recently, rotavirus A infection in Chiang Rai Province peaked from March to May of 2019 to 2020 [38]. Altogether, most diarrheal cases of rotavirus infection tend to appear in the cool or dry months of the year in Thailand.

### 3.3. Distribution of Rotavirus A Genotypes in Pediatric Patients

**Figure 2:**
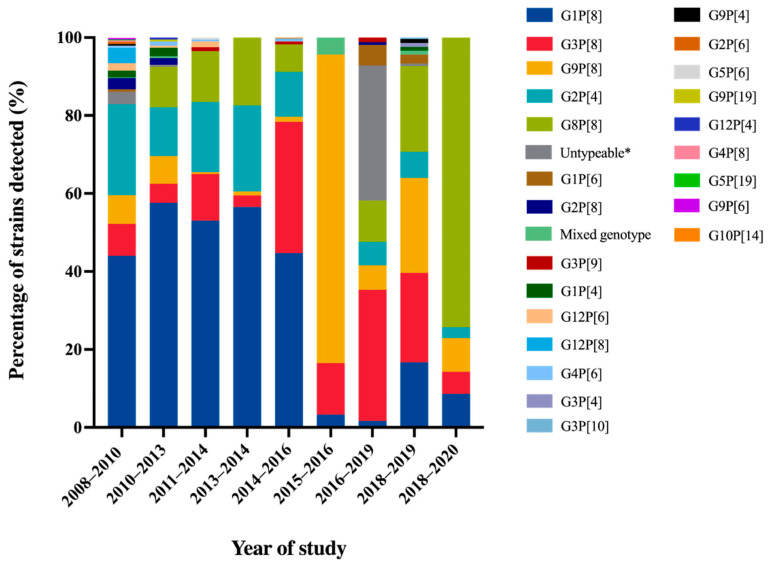
Distribution of Rotavirus A Genotypes in Pediatric Patients with Diarrhea in Thailand from 2008 to 2020. * No amplicon/no PCR product/unsuccessful sequencing.

A comprehensive review article describing rotavirus A prevalence in children suffering from acute gastroenteritis in Thailand between 2000 and 2007 revealed the identification of a wide range of rotavirus genotypes. These genotypes included G1P[8], G2P[4], G2P[8], G3P[3], G3P[8], G3P[9], G3P[10], G3P[19], G9P[8], G12P[6], and G12P[8]. Notably, among these genotypes, G1P[8] and G9P[8] emerged as the predominant circulating strains within this country [16]. Molecular epidemiological studies of rotavirus G and P genotypes distribution in Thailand over twelve years from 2008 to 2020 are summarized in Figure 2. The rotavirus G1P[8] genotype was still detected as the most dominant genotype in Thailand over 40% from 2008 to 2016. Then, it decreased dramatically to less than 6.1% during the study period of 2015 to 2020 [33,38]. Similarly, the trend of G2P[4] detection was also downward from 11.5–23.3% from 2008 to 2016 to 6.0–6.7% from 2016 to 2019. On the contrary, G9P[8] and G3P[8], which were the minor rotavirus genotypes detected over seven years period from 2008 to 2014, increased sharply to the most prevalent genotype from 2015 to 2019. Interestingly, G8P[8] became the most prevalent genotype from 2018 to 2020 [38]. The emergence of G8P[8] as the most predominant genotype with an exceptionally high frequency in Chiang Rai has never been observed before in this area. It is noteworthy that the predominance of G8P[8] genotypes in Chiang Rai province aligns with a study conducted in Chiang Mai province during the same period, from 2018 to 2019 [39]. These findings strongly suggest that there has been a significant change in the circulating strains of rotavirus in Thailand over time. Furthermore, the uncommon rotavirus strains, G1P[4], G1P[6], G2P[6], G2P[8], G3P[4], G3P[9], G3P[10], G4P[6], G4P[8], G5P6], G5P[19], G9P[4], G9P[6], G9P[19], G10P[14], G12P[4], G12P[6], G12P[8], were detected periodically at low frequency in Thailand. Some of these strains have been characterized and reported as animal-like rotaviruses strains, for instance, feline-like G3P[9], porcine-like G4P[6], bovine-like G8P[8], and bat-like G3P[10] human rotavirus strains [33,40].

Overall, the most common rotavirus A genotypes that circulated in Thailand over twelve years period from 2008 to 2020 were G1P[8], G2P[4], G3P[8], G8P[8], and G9P[8] genotypes. The changing of rotavirus genotypes has also been observed in other countries, especially in Southeast Asian countries. The most predominant genotype has changed from G1P[8] to the G3P[8], G8P[8], and G9P[8] genotypes [44]. This phenomenon could be attributable to the acquisition of immunity previously exposed and lacking herd immunity against that genotype. In addition, rotavirus vaccines, RotaTeq and Rotarix, were licensed as optional vaccines in Thailand in 2005 and 2008, respectively, [9,45] and possibly impacted the alteration of rotavirus predominant genotypes.

### 3.4. Prevalence and Distribution of Rotavirus A Genotypes in Animals 

Rotavirus A is a diarrheal pathogen that can infect a broad range of animal species, such as pigs, cows, dogs, and cats [44]. Rotavirus A genotypes that have been detected in pigs worldwide are thirteen G genotypes (G1 to G6, G8 to G12, and G26) and seventeen P genotypes (P[1] to P[8], P[11], P[13], P[19], P[23], P[26], P[27], P[32], P[34], and P[49]) [47]. The most common G genotypes in pigs are G3, G4, G5, G9, and G11, in associated with P[6], P[7], P[13], P[19], and P[23] [48]. In cattle, at least 12 G types (G1–G3, G5, G6, G8, G10, G11, G15, G17, G21, and G24) and 11 P types (P[1], P[3], P[5-7], P[11], P[14], P[17], P[21], P[29], and P[33]) have been reported. However, the most prevalent genotypes in calves are G6, G8, and G10, as well as P[1], P[5], and P[11] [12]. The most common rotavirus A genotypes in canines are G3P[3] and G3P[9], whereas G3P[3] and G3P[9] are the most prevalent genotypes in felines [49].

The distribution of rotavirus in various animal species in Thailand from 2011 to 2023 is summarized in Table 2. In Thailand, few published articles on rotavirus A infection in animals exist. Most studies reported the detection of rotavirus A in bovine and porcine species from 1988 to 2010 [16]. Likewise, one study conducted the surveillance of rotavirus A in pigs in Chiang Mai and Lamphun Provinces from 2011 to 2014 [50]. It was observed that the prevalence of rotavirus A infection in piglets under four weeks old was 23.0%, and the most predominant genotype was G4P[13] (29.2%), followed by G4P[23] (14.1%), G5P[23] (11.5%), G4P[6] (9.7%), G3P[23] (7.0%), G5P[13], (6.1%), G3P[13] (4.4%), G3P[6] (2.7%), and G5P[6] (2.7%). Moreover, G3P[19], G4P[7], G9P[19], G9P[23], G9P[7], G4P[19], and G11P[13] genotypes were also detected at a low percentage. Another study conducted the surveillance of porcine rotavirus infection in diarrheic piglets across seventeen provinces in the Northeast, East, Central, West, and South of Thailand from 2011 to 2016 [51] and found that the prevalence of porcine rotavirus infection was 9.5%. Then, 24 porcine rotavirus strains from the former study were randomly identified with G and P genotypes [52]. The dominant genotype in piglets was G9P[13] (25.0%) and G9P[23] (25.0%), followed by G3P[13] (20.8%), G9P[19] (12.5%), G4P[6] (8.3%), G4P[19] (4.2%), and G5P[23] (4.2%). In addition, the prevalence of canine rotavirus infection was found at 0.7% in dogs under one-year-old in 2016 to 2019 from five provinces, including Nakhon Si Ayutthaya, Bangkok, Suphan Buri, Nakhon Rachasima, and Tak, and all of them were G3P[3] genotypes [53]. Recently, the G3P[9] rotavirus strain was detected in a two-year-old female cat with severe diarrhea in Bangkok in 2021 [54]. Overall, the prevalence and predominant strains of rotavirus A in domestic animals in Thailand align with the findings from other studies [55,56,57].

## 4. Interspecies Transmission of Rotaviruses

Rotaviruses are constantly changing, and most of these changes are driven by interspecies transmission and reassortment of segmented rotavirus A genomes, which are important mechanisms of rotavirus evolution leading to the great diversity of human rotavirus A [12,14]. In Thailand, numerous studies reported the detection of unusual rotavirus strains bearing the gene segments derived from the rotaviruses of both human and animal origins. For evidence of interspecies transmission of porcine rotavirus to humans, the G9P[23] was detected in a ten-month-old child hospitalized with severe diarrhea, and this uncommon rotavirus strain carried non-G/P human rotavirus genome with the constellation of I5-R1-C1-M1-A8-N1-T1-E1-H1, which was similar to those of porcine rotavirus strains [58]. In addition, the surveillance of rotavirus infection in children admitted to the hospitals with diarrhea in Udon Thani and Chanthaburi provinces revealed that two G4P[6] strains exhibited a Wa-like genotype constellation, except for the NSP1 genotype of A8 that is generally found in porcine rotavirus strains [59]. Based on full genome constellation and phylogenetic analyses, two rare human G4P[6] rotavirus strains that contained the genotype constellation of porcine G4P[6] were also detected in patients with diarrhea in Chiang Mai, and they were most similar to the G4P[6] porcine rotavirus strains detected previously in the same geographical area [60]. Moreover, a rare genotype of G5P[19] was first detected in an asymptomatic patient and displayed a close genetic relationship with porcine rotavirus strains detected from 2008 to 2010 [32]. The occurrence of zoonotic transmission of a bovine rotavirus strain was also frequently identified in Thailand, such as G6P[14] and G10P[14] human rotavirus strains detected from children with diarrhea carrying the genetic backbone of I2-R2-C2-M2-A3-N2-T6-E2-H3 similar to those of bovine rotavirus strains [61,62]. Furthermore, Chieochansin et al. [34] reported the detection of several rotaviruses with unusual genotypes, including one feline-like G3P[9], one bovine-like G8P[8], and four porcine-like (G4P[6], G5P[6], G9P[8], and G12P[6]) human rotavirus strains, suggesting a great diversity of animal-like rotavirus strains circulating in children with diarrhea in Thailand.

The decrease in the detection of common human rotavirus G1P[8] with Wa-like and the increase in the detection of unusual human rotavirus DS-1-like reassortant strains recently were observed in Thailand. From 2010 to 2013, G8P[8] strains with DS-1-like genotype constellation were detected at high frequency in Chiang Mai Province, Northern Thailand. The genome sequence analysis of these G8P[8] strains revealed that they were closely related to bovine rotavirus and bovine-like human rotavirus strains [33]. Moreover, G9P[19] rotavirus strains detected in children with diarrhea in Chiang Mai Province showed genome sequences closely related to those of G9P[19] porcine rotavirus strains isolated in the same area. These findings imply that interspecies transmission among porcine and human rotaviruses occurred in nature [63]. From 2013 to 2014, Tacharoenmuang et al. [64] reported the detection of novel DS-1-like G8P[8] reassortant strains which carried both human and bovine rotavirus gene segments in stool specimens of children hospitalized with severe diarrhea. 

The surveillance of rotavirus infection in Thailand from 2012 to 2014 revealed that the uncommon DS-1-like G1P[8] strains carrying DS-1-like human rotavirus genes (G1-P[8]-I2-R2-C2-M2-A2-N2-T2-E2-H2) were detected and G1P[8] appeared to be originated from human Wa-like G1P[8] strains through reassortment and these Thai G1P[8] strains were most similar to the Japanese DS-1-like G1P[8] [65]. The finding implied that the DS-1-like G1P[8] strains isolated from Thailand and Japan originated from the same ancestor. 

Rotavirus is one of the viral pathogens that infect many host species, including bats. In the last decade, the bat has been the potential source for emerging rotaviruses in humans and is considered to be the reservoir for many other emerging viruses [66,67]. In Thailand, there are sporadic reports of bat-like human rotavirus strains. Up To date, four published articles have reported the detection of G3P[10] bat-like human rotavirus strains in Thailand [39,67,68,69]. The first G3P[10] bat-like human rotavirus strain (CMH079) was isolated from a two-year-old child admitted to the hospital with diarrhea in Chiang Mai in 2005 [69]. Ten years later, in 2015, the bat-like G3P[10] (MS2015-1-0001) was detected in an eleven-month-old child hospitalized with acute gastroenteritis in Tak Province. This atypical G3 strain exhibited high nucleotide sequence identity with Thai human DS-1-like G3P[8] (PK2015-1-0037) and Chinese bat rotavirus (MYAS33) strains [67]. From 2016 to 2018, there was an additional report of the detection of two uncommon G3P[10] rotavirus strains from patients with diarrhea, and these strains were also highly similar to Chinese bat rotavirus MYAS33 and Thai bat-like human rotavirus MS2015-1-0001 strains. Interestingly, one of the G3P[10] strains in this study has a unique genome constellation (G3-P[10]-I3-R3-C3-M3-A9-N3-T3-E3-H6), which has not been reported in the literature before [68]. In 2019, the most recent study reported that the unusual G3P[10] (CMH-S015-19) rotavirus strain had been identified from a diarrheic one-year-old child in Chiang Mai [39]. Phylogenetic analysis of VP7 and VP4 genes of CMH-S015-19 strain showed a close genetic relationship with those of bat rotavirus strains (LUS12-14 and MYAS33) and also similar to the G3P[10] (CMH079) human rotavirus strain, which was isolated in the same area 15 years ago [69]. Increasing interaction between humans and wild animals such as bats leads to an elevated risk of zoonotic rotavirus transmission.

## 5. Conclusions and Further Perspectives

In Thailand, the accumulation of data on the prevalence, seasonal pattern, and genetic diversity of rotaviruses has been reported continuously for almost four decades. Rotavirus has been recognized as the major viral causative agent of acute gastroenteritis in children younger than five years of age. The rotavirus G1P[8] genotype was detected as the most predominant genotype and abruptly decreased from 2017 to 2020. In contrast, recently, other genotypes such as G9P[8], G3P[8], and G8P[8] have been detected as the predominant genotypes. In addition, the interspecies transmission of rotavirus strains has been occasionally detected in Thailand, such as G3P[10], G4P[6], and G9[P23]. Most recently, rotavirus vaccines were introduced as part of the national childhood immunization program in Thailand in 2020. Further epidemiological surveillance of rotavirus infection after the implementation of rotavirus vaccines is needed to be conducted to investigate the influence of rotavirus vaccines on the prevalence, seasonality, and diversity of rotavirus strains circulating in Thailand.

## Figures and Tables

**Table 1:** Prevalence of Human Rotavirus A Infection in Children Hospitalized with Acute Gastroenteritis in Thailand.

Year of Study	Place of Study	No. of Specimens Tested	No. of Rotavirus A Positive (%)	Detection Method	Age	Age Group with High Infection Rate	Seasonal Pattern	References
2008–2010	Chiang Rai, Nakhon- Ratchasima, Surat Thani, Phitsanulok	3470	458 (26.8)	qRT-PCR, ELISA	3–60 months	<2 years	January–March (52.0–69.0%)	[32]
2010–2013	Chiang Mai	1032	184 (17.8)	RT-PCR	0–15 years	12–24 months (54%; 100 of 184)	-	[33]
2011–2014	Bangkok, Khon Kaen	688	204 (30.0)	RT-PCR	<15 years	1–5 years	January–May	[34]
2013–2014	Phechaboon, Sukothai	2754	666 (24.2)	PAGE	-	-	-	[35]
2014–2016	Bangkok, Udonthani, Beung Kan, Phuket, Tak, Chanthaburi	1867	514 (27.5)	RT-PCR	0–>60 years	5–10 years (43%; 59 of 137)	November–April	[36]
2015–2016	Chiang Rai	1867	91 (33.7)	Multiplex RT-PCR	0–5 years	12–23 months (45.1%; 41 of 91)	March (64.8%)	[37]
2016–2019	Bangkok	2001	301 (15.0)	qRT-PCR,	0–<15 years	0–24 months	December–March	[40]
2018–2019	Chiang Mai	1170	209 (17.9)	Nested RT-PCR	0–5 years	-	January–March (39.8–63.3%)	[39]
2018–2020	Chiang Rai	302	35 (11.5)	qRT-PCR	<5 years	6–11 months (37.1%; 13 of 35)	March–May	[38]

ELISA, enzyme-linked immunosorbent assay; RT-PCR, reverse transcription-polymerase chain reaction; qPCR, real-time polymerase chain reaction; PAGE, polyacrylamide gel electrophoresis.

**Table 2:** Distribution of Rotavirus Infection in Various Animal Species in Thailand, 2011 to 2023.

Year of Study	Place	Hosts	No. of Specimens Tested	No. of Rotavirus A Positive (%)	Detection Method	Age of Animals	Age Group with High Infection Rate	References
2011–2014	Chiang Mai, Lamphun	Piglets	491	113 (23.0)	RT-PCR	0–4 weeks	-	[50]
2011–2016	Kanchanaburi, Prachuap Khiri Khan, Phechaboon, Ratchaburi, Lop Buri, Samut Songkhram, Suphan Buri, Saraburi, Phra Nakhon Si Ayutthaya, Nakhon Pathom, Chon Buri, Chachoengsao, Ubon Ratchathani, Udon Thani, Nakhon Ratchasima, Trang, and Nakhon Si Thammarat	Piglets	769	73 (9.5)	RT-PCR	0–>12 weeks	≥4–8 weeks	[51]
2016–2019	Nakhon Si Ayutthaya, Bangkok, Suphan Buri, Nakhon Rachasima, Tak	Dogs	710	5 (0.7)	RT-PCR	0–>1 years	<1 year	[53]
2021	Bangkok	Cat	1 *	1 (100.0)	qRT-PCR	2 years	-	[54]

* The specimen was obtained from a two-year-old female Siamese pet cat with bloody mucoid diarrhea at a local private clinic in Bangkok in 2021.
